# Analyzing Information Exchange in Parkinson’s Disease via Eigenvector Centrality: A Source-Level Magnetoencephalography Study

**DOI:** 10.3390/jcm14031020

**Published:** 2025-02-05

**Authors:** Michele Ambrosanio, Emahnuel Troisi Lopez, Maria Maddalena Autorino, Stefano Franceschini, Rosa De Micco, Alessandro Tessitore, Antonio Vettoliere, Carmine Granata, Giuseppe Sorrentino, Pierpaolo Sorrentino, Fabio Baselice

**Affiliations:** 1 Department of Economics, Law, Cybersecurity and Sports Sciences (DiSEGIM), University of Naples “Parthenope”, 80035 Nola, Italy; michele.ambrosanio@uniparthenope.it (M.A.); giuseppe.sorrentino@uniparthenope.it (G.S.); 2 Department of Education and Sport Sciences, Pegaso Telematic University, 80143 Naples, Italy; e.troisilopez@gmail.com (E.T.L.); carmine.granata@isasi.cnr.it (C.G.); 3 Department of Engineering, University of Napoli “Parthenope”, 80143 Napoli, Italy; mariamaddalena.autorino001@studenti.uniparthenope.it (M.M.A.); stefano.franceschini@studenti.uniparthenope.it (S.F.); fabio.baselice@uniparthenope.it (F.B.); 4 Department of Advanced Medical and Surgical Sciences, University of Campania “Luigi Vanvitelli”, 81100 Naples, Italy; rosa.demicco@unicampania.it (R.D.M.); alessandro.tessitore@unicampania.it (A.T.); 5 Institute of Applied Sciences and Intelligent Systems, National Research Council, 80078 Pozzuoli, Italy; a.vettoliere@isasi.cnr.it; 6 ICS Maugeri Hermitage Napoli, via Miano, 80145 Naples, Italy; 7 Institut de Neurosciences des Systèmes, Aix-Marseille Université, 13007 Marseille, France; 8 Department of Biomedical Sciences, University of Sassari, 07100 Sassari, Italy

**Keywords:** Parkinson’s disease, magnetoencephalography, eigenvector centrality, brain network

## Abstract

**Background:** Parkinson’s disease (PD) is a progressive neurodegenerative disorder that manifests through motor and non-motor symptoms. Understanding the alterations in brain connectivity associated with PD remains a challenge that is crucial for enhancing diagnosis and clinical management. **Methods:** This study utilized Magnetoencephalography (MEG) to investigate brain connectivity in PD patients compared to healthy controls (HCs) by applying eigenvector centrality (EC) measures across different frequency bands. **Results:** Our findings revealed significant differences in EC between PD patients and HCs in the alpha (8–12 Hz) and beta (13–30 Hz) frequency bands. To go into further detail, in the alpha frequency band, PD patients in the frontal lobe showed higher EC values compared to HCs. Additionally, we found statistically significant correlations between EC measures and clinical impairment scores (UPDRS-III). **Conclusions:** The proposed results suggest that MEG-derived EC measures can reveal important alterations in brain connectivity in PD, potentially serving as biomarkers for disease severity.

## 1. Introduction

Parkinson’s disease (PD) is a disabling neurodegenerative disorder traditionally regarded mainly as a motor disease. However, supported by clinical and neuroimaging findings, this view has been largely questioned [1]. For example, structural Magnetic Resonance Imaging (MRI) studies have shown that the regions involved in PD are more widespread than previously thought. Functional magnetic resonance imaging (fMRI) studies have also identified changes in functional connectivity that extend to many other brain systems well beyond the corticostriatal network [2]. However, the multifaceted nature of large-scale brain interactions in PD is highlighted by contrasting reports, with studies reporting more-connected [3], less-connected networks, and a combination of both [4].

Besides fMRI, relevant information on the functioning of the brain networks can be obtained using neurophysiological techniques, such as electroencephalography (EEG) and magnetoencephalography (MEG), since they directly capture the electrical/magnetic activity of the neuronal ensembles [5]. MEG is a non-invasive neuroimaging technology that measures the magnetic fields produced by neuronal activity providing high temporal resolution and sensitivity to the dynamics of oscillatory brain activity across different frequency bands. This makes it a valuable tool for investigating functional connectivity and neural network alterations in health and disease [6]. In the context of PD, MEG has been employed to explore changes in brain oscillatory activity, particularly in the alpha and beta frequency bands, which are known to be associated with motor control and cognitive functions [7,8]. MEG studies have also reported widespread alterations in multiple functional connections [9]. Finally, taking advantage of the high temporal resolution of MEG, recent studies, specifically addressing the dynamical changes in PD, demonstrated that patients showed stereotypical brain dynamics and reduced flexibility, and that the intensity of this reduction was proportional to symptom severity.

In recent years, to extract relevant features of the interactions among cerebral areas, brain networks have been characterized using graph theory, a mathematical approach to represent complex interactions among multiple elements [10]. To enable characterization of brain network topology using graph analysis, brain areas are typically used as nodes and the interactions between brain areas as links. However, comparing the results obtained by using this approach is non-trivial, as they are influenced by network size, thresholding or edge density, not allowing for a purely topological interpretation of the results [11].

Eigenvector centrality (EC) is a graph theory-based metric that quantifies the importance of a node within a network, considering both the number and quality of its connections, and both the node’s direct connections and the connections of its neighbors [12,13,14,15]. This measure is particularly useful for identifying key hubs in the brain that may play a crucial role in maintaining network integrity and functionality. EC offers distinct advantages over other graph theory-based metrics. Unlike degree centrality, which only accounts for the number of direct connections of each node, EC considers both the quantity and the quality of these connections by evaluating the influence of a node within the context of the entire network. This provides a more holistic view of a node’s importance, particularly useful in understanding complex brain networks. In comparison to betweenness centrality, another metric largely used, which focuses on the number of shortest paths passing through a node, EC provides a continuous measure of node influence that captures the cumulative effects of indirect connections. This is advantageous for identifying key hubs in the brain that may not necessarily lie on many shortest paths but still play a crucial role in maintaining network integrity and functionality. Finally, the clustering coefficient and the local efficiency measure local connectivity patterns, but do not provide insights into the global network structure as EC does. EC bridges this gap by reflecting both local and global network properties, making it an interesting metric for investigating the widespread and complex connectivity alterations as observed in PD. Thus, by applying EC to MEG data, it is possible to identify key brain regions that play a central role in the overall network, potentially uncovering biomarkers of disease states [16].

In this study, we employed EC measures derived from MEG data to investigate possible alterations in brain connectivity in individuals with PD compared to healthy controls (HCs). Additionally, we decided to explore the potential involvement of specific brain regions beyond the conventional motor areas traditionally associated with the pathology, finding results partially aligned with [13]. Relatively to the acquisition step, MEG data were collected according to [17,18]. Functional connectivity matrices were constructed for each participant by adopting a metric named phase linearity measurement [19,20,21], which is insensitive to volume conduction and resilient to noise, and EC measures were computed for different brain regions. More specifically, we focused on five lobes (e.g., frontal, insular, temporal, parietal, and occipital lobe), and subcortical regions. Statistical analyses were then performed to compare EC values between PD patients and HCs. Finally, we investigated possible correlations between EC values and clinical impairment scores.

## 2. Materials and Methods

### 2.1. Participants

A total of 47 individuals with Parkinson’s disease (PD)—30 men and 17 women—were included in this study. Their mean age was 65 ± 9.7 years, and they had an average of 11.3 ± 4.2 years of education. Parkinson’s disease was diagnosed according to the United Kingdom Parkinson’s Disease Brain Bank criteria [22]. To be enrolled, participants had to (a) show onset of PD after 40 years of age (to exclude early-onset cases) and (b) have a modified Hoehn and Yahr (H&Y) stage of 2.5 or lower [23]. Exclusion criteria were (a) dementia associated with PD, defined according to established [24], and (b) any other neurological disorder or significant medical condition. Notably, no individual met McKeith et al. [25] criteria for Lewy body dementia. Moreover, none of the participants displayed focal slowing or had a history of epilepsy or seizures. Disease severity was evaluated in the off-medication state using both the UPDRS-III [26] and H&Y staging, with an average H&Y score of 1.83, indicating that the PD cohort consisted of patients in the early stages of the disease. Further details on these individuals are provided in Table S1. The patients made no continuous use of benzodiazepines or having used them at any point in the two weeks prior to the MEG recording.

In addition, 47 healthy controls (HCs) matched for sex (30 men, 17 women), age (61.8 ± 10 years), and education (12.9 ± 4.6 years) were recruited. All participants were right-handed. This study was conducted in accordance with the Declaration of Helsinki, and each participant provided informed consent. Approval for this study was obtained from the local Ethics Committee of the University of Naples “L. Vanvitelli”.

### 2.2. MEG Acquisition

The magnetoencephalography (MEG) data were collected using a 154-channel system developed by the Institute of Applied Sciences and Intelligent Systems at the Italian National Research Council [27]. This device employs highly sensitive superconducting quantum interference device (SQUID) magnetometers, arranged in a helmet-shaped configuration to encompass the entire head. The SQUIDs are maintained at liquid helium temperature (4.2 K) inside a high-performance fiberglass dewar, which both minimizes additional magnetic noise to levels comparable to the sensors and ensures low heat transfer. As a result, the system can operate for approximately one week on a single helium refill. The SQUIDs are positioned just 2 cm from the outside surface of the dewar thanks to efficient thermal insulation, and neighboring sensors are spaced 3 cm apart.

Because brain-generated magnetic signals are exceedingly small—on the order of eight magnitudes lower than typical environmental interference—the entire setup is housed in a magnetically shielded room (MSR). This MSR comprises two layers of high-permeability (μ-metal) material to attenuate low-frequency noise and a single aluminum plate to reduce high-frequency electromagnetic interference by means of eddy currents. Overall, the room achieves a shielding factor of 35 dB at 10 mHz and 100 dB at 20 Hz. The SQUID magnetometers themselves are fabricated on 8 × 8 mm^2^ superconducting chips, where an external field induces screening current in a pick-up coil connected to a multiturn input coil. This input coil is inductively coupled to a superconducting loop interrupted by two Josephson junctions (bare SQUID), translating magnetic flux into a voltage output. Through the combination of high-quality Josephson junctions and a dedicated feedback circuit, the system achieves a magnetic field noise level of approximately 2–3 fT/√Hz, with low-frequency noise remaining below a few Hz. The feedback coil, designed in a bipolar shape and integrated into the SQUID chip, further minimizes cross-talk between adjacent channels [28].

Before each recording, head reference points were obtained using a Fastrak (Polhemus) system [29], allowing precise determination of head position during data acquisition. Participants were instructed to remain at rest with eyes closed throughout the procedure. Individuals with Parkinson’s disease (PD) were recorded in the “off-state”, following a 14 h washout from medication. Two consecutive runs—each lasting 3.5 min—were conducted, with a brief two-minute interval in between to mitigate fatigue or drowsiness [30,31]. Additionally, electrocardiographic (ECG) and electro-oculographic (EOG) signals were captured for artifact removal [31]. All signals were sampled at 1024 Hz and underwent anti-aliasing filtering prior to digitization.

### 2.3. MRI Acquisition

A total of 80 participants underwent magnetic resonance imaging (MRI) using a 1.5 T SIGNA Explorer scanner (GE Healthcare, Milwaukee, WI, USA) equipped with an eight-channel head coil. Three-dimensional T1-weighted images were obtained via a gradient-echo inversion recovery sequence. The main acquisition parameters included repetition time (TR) = 8.216 ms, inversion time (TI) = 450 ms, echo time (TE) = 3.08 ms, flip angle = 12°, voxel size = 1 × 1 × 1.2 mm^3^, and a 256 × 256 matrix. Of the initial pool of 94 enrolled individuals, 7 patients and 7 controls were unable or unwilling to undergo MRI; consequently, a standard template was applied for source reconstruction in these cases.

### 2.4. MEG Processing and Source Reconstruction

Data preprocessing and source reconstruction were carried out in accordance with the method outlined in [32]. In brief, the signals were band-pass filtered between 0.5 and 48 Hz using a fourth-order Butterworth IIR filter in the Fieldtrip toolbox for MATLAB [33]. Next, principal component analysis (PCA) was used to orthogonalize the MEG signals relative to reference signals [34]. An experienced researcher examined the recordings to remove any segments contaminated by noise. Subsequently, a supervised independent component analysis (ICA) approach was employed to identify and eliminate cardiac (ECG) and, when present, ocular (EOG) artifacts [35].

**Figure 1:**
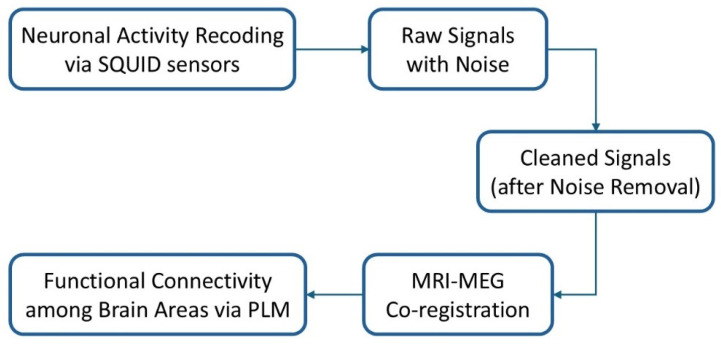
Details of the MEG data processing pipeline. The neuronal activity is recorded via 154 SQUID sensors; then, the raw signal is filtered from the noise (e.g., cardiac activity and blinking artifacts). The cleaned signal is then co-registered with the MRI signal of each subject to obtain the source activity reconstruction. Finally, the functional connectivity among brain areas is estimated per each of the 90 brain areas and per each frequency band (e).

The MEG data were then co-registered with each participant’s MRI. Time series were extracted for 116 regions of interest (ROIs) defined by the AAL atlas [36,37] using Nolte’s volume conduction model [Nolte, 2003] and the linearly constrained minimum variance (LCMV) beamformer. These time series were further divided into standard frequency bands—delta (0.5–4 Hz), theta (4–8 Hz), alpha (8–13 Hz), beta (13–30 Hz), and gamma (30–48 Hz). For the subsequent analyses, only 90 ROIs were retained, as cerebellar regions were excluded due to lower reliability. Synchronization between brain areas was quantified via the phase linearity measurement [19]. A flowchart of the overall pipeline is shown in Figure 1.

To minimize the risk of both type I and type II errors, and to align with the study hypothesis, the eigenvector centrality (EC) values were averaged within each lobe, resulting in one EC value for each of the five lobes (frontal, insular, temporal, parietal, and occipital) as well as one for subcortical regions. Further details of this partitioning are provided in the Supplementary Material.

### 2.5. Eigenvector Centrality

In graph theory, eigenvector centrality (EC), also referred to as eigencentrality or prestige score [38], evaluates the influence of a node within a connected network. Each node’s score is determined by taking into account the scores of the nodes to which it is connected; in other words, having links to nodes with high scores increases a node’s EC more than having links to nodes with lower scores. Therefore, a node with a high eigenvector centrality is typically connected to numerous other nodes that themselves hold high centrality values [39,40].

Let us consider a graph G ≔ (V,E), where V is the set of vertices (or nodes), and E is the set of edges (or links). We define $A=\left(a_{i,j}\right)$ as the connectivity matrix, where $a_{i,j}$ captures the strength of the connection between vertices i and j, with values spanning the interval [0, 1]. The relative centrality score $x_{i}$ of vertex i can be expressed as

(1) $$x_{i}=\frac{1}{\lambda }\sum_{t\;\epsilon \;N(i)}x_{t}=\frac{1}{\lambda }\sum_{t\;\epsilon \;V}{a_{i,t}\;x}_{t},$$

in which N(i) is the set of neighbors of i and λ is a constant. Equation (1) can be re-arranged in a vector notation as eigenvector equation

(2) Ax=λx,

The solution to Equation (2) is the eigenvector x corresponding to the largest eigenvalue λ of the matrix A. The EC scores are normalized so that the sum of the squares of the scores is 1 (i.e., the vector x has unit length). This normalization helps in comparing the centrality scores across different networks or nodes. The normalized EC score can be computed as

(3) $${\tilde{x}}_{i}=\frac{x_{i}}{\sqrt{\sum_{j=1}^{N}x_{j}^{2}}}$$

This normalization ensures that the centrality scores are scaled proportionately and provides a standardized way to interpret the influence of each node within the network, i.e., nodes with high EC scores are those that are connected to many nodes who themselves have high scores, indicating their strategic position within the network.

### 2.6. Statistical Analysis

To identify potential significance between the two populations of PD and HCs, permutation tests were carried out. Specifically, per each comparison, the *p*-value was obtained by comparing the absolute difference in the actual means of the two groups to the distribution of surrogate absolute differences obtained by randomly shuffling the observations across groups at each of the 50,000 iterations. The results were corrected by adopting the Benjamini–Hockberg procedure (BH step-up procedure) [41] to control the false discovery rate (FDR) at level 0.05. These tests were conducted on a population of 47 subjects. Subsequently, Pearson’s correlation was used to find possible correlations between EC and clinical scales (i.e., disease duration and UPDRS-III).

## 3. Results

**Figure 2:**
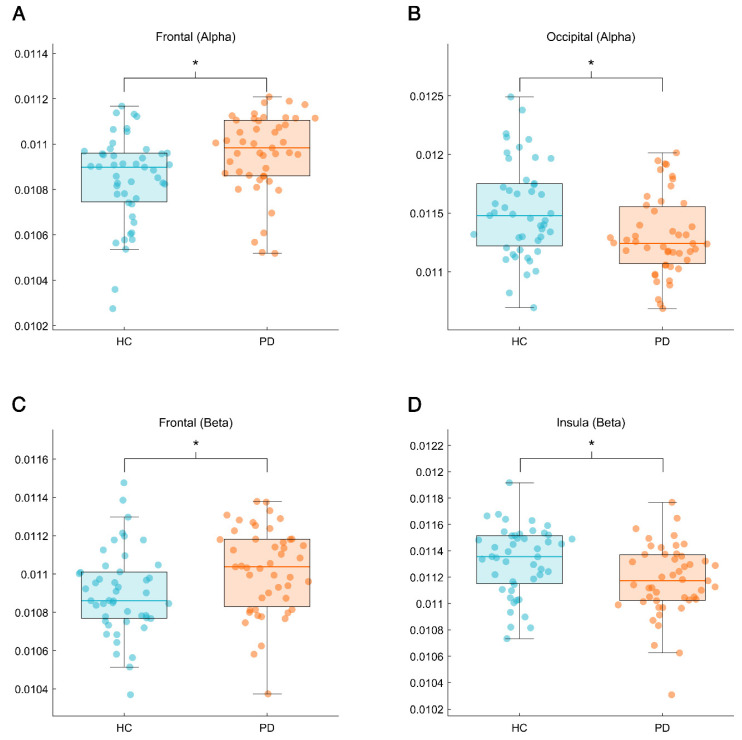
Results of permutation test (number of permutations: 50,000). The four panels display the comparison between eigenvector values of patients with Parkinson’s disease (PD) and healthy controls (HCs) in different lobes and different bands. In particular, panel (**A**) displays a significant difference in the frontal lobe in alpha band; panel (**B**) displays a significant difference in the occipital lobe in beta band; panel (**C**) shows a significant difference in the frontal lobe in beta band; and panel (**D**) shows a significant difference in the insula in beta band. Significance *p*-value is set at *p* < 0.05 after false discovery rate correction and referred to with the asterisk symbol (*) at the top of the subfigures.

The EC values of the five lobes and subcortical brain regions were compared between PD patients and HCs, and a correlation analysis between this measure and the clinical scale and the duration of the disease was performed within the patients’ group. This analysis was performed per each frequency band. As a first step, permutation tests were performed (number of permutations: 50,000) to find statistically significant differences between the two populations. As reported in Figure 2, the bands with some statistically significant differences were the alpha and beta bands.

To go into further detail, it was observed that the PD population showed higher values of EC relatively to the frontal lobe with respect to HCs in both alpha (p_FDR_ = 0.014) and beta band (p_FDR_ = 0.026), as shown in Figure 2. Conversely, the occipital lobe was characterized by lower values of EC (p_FDR_ = 0.014) for the PD patients with respect to HCs in the alpha band, and the same trend was observed for the EC values for the insula lobe (p_FDR_ = 0.034) in the beta band (Figure 2). Furthermore, a trend towards a statistically significant difference between the two populations was observed for the subcortical regions (not reported in the figures).

We also checked whether the same results were confirmed when comparing the populations separately by sex. In the male population (*n* = 30), the results were confirmed in the alpha band (frontal lobe p_FDR_ = 0.003; occipital lobe p_FDR_ = 0.014), while in the beta band, only the frontal lobe showed a trend toward significance before correction (*p* = 0.052). In the female population (*n* = 17), however, the results were not confirmed. 

**Figure 3:**
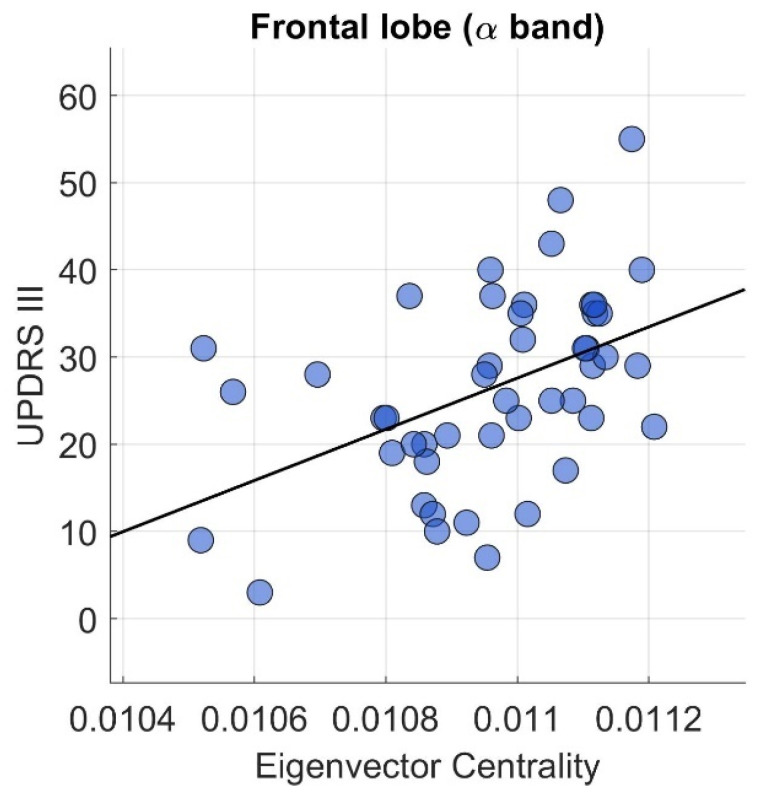
Results using Pearson correlation between EC measure and the clinical scale of UPDRS-III on PD protocol for the frontal lobe in alpha band (r = 0.46, p_FDR_ = 0.007).

In addition, a correlation analysis between EC in the brain area that showed a difference as compared to controls and clinical parameters such as the clinical UPDRS-III scale and the duration of the disease, was carried out. Figure 3 shows the Pearson correlation plot for the case which presented correlation that is statistically relevant after correction. In the frontal lobe, a direct correlation in the alpha band was observed between EC measure and the disability clinical scale of UPDRS-III (r = 0.46, p_FDR_ = 0.007) (Figure 3) before correction of a similar correlation (same area and frequency band) was observed between EC and disease duration (r = 0.33, *p* = 0.025). Furthermore, a statistically significant correlation before correction (r = 0.30, *p* = 0.040) between UPDRS-III scale and EC measure was observed in the beta band. Finally, in the occipital lobe, a significant correlation before correction (r = −0.32, *p* = 0.033) with the UPDRS-III scale was observed in the alpha band. Correlation plots are reported in Supplementary Materials (Figure S1).

## 4. Discussion

This study has two primary objectives. The first is to determine whether the topological measure of eigenvector centrality (EC) can effectively capture connectivity changes that have clinical relevance in PD. The second objective is to identify the specific brain lobes where these potential changes in EC occur. Brain network topological alterations in PD patients have been widely reported using different approaches and topological metrics, often leading to conflicting results [42,43,44,45,46]. In this wide framework, our findings highlight significant distinctions in alpha and beta frequency bands in PD patients as compared to HCs. We observed that both in alpha and beta band in the frontal pole of PD patients, EC values were higher than in controls. Furthermore, EC in alpha band correlated directly with motor disability assessed by UPDRS III. In other words, the higher the EC, the more clinically compromised the patients were. The clinical significance of this correlation found further support in the fact that EC also correlated positively with disease duration. However, it should be noted that after FDR correction the statistical significance was lost, probably due to the relatively small sample size. In addition, in comparison to other graph theory-based metrics, eigenvector centrality provides distinct advantages, making it particularly valuable for the analysis of PD. While metrics like degree centrality or betweenness centrality offer insights into local or specific aspects of network connectivity, EC captures the global importance of a node within the entire network. This ability to reflect both direct and indirect connections makes EC a powerful tool for identifying key hubs that are crucial for maintaining network integrity.

Our study reveals higher median EC in the alpha and beta bands within the frontal lobes of PD patients compared to HCs. The frontal lobe is integral to executive and motor functions, whose impairment is a common finding in PD [47]. For instance, in [44] the authors revealed significant decreases in brain gray matter regions in the medial frontal gyrus and supplement motor area in PD patients compared to HCs, indicating topological changes in the frontal lobe. In [48], the authors highlight abnormalities in degree centrality in the right inferior frontal gyrus and the caudal middle frontal lobe, which were linked to cognitive scores in PD patients. The study proposed by Li et al. [43] found widespread topological changes in the frontal and parietal regions in PD patients, including increased shortest path length and decreased global efficiency [49]. It must be noted that the patients included in the study were assessed in an off-medication state, meaning they were not taking levodopa at the time of the recordings. This is particularly relevant as levodopa is known to affect beta activity [50,51] in regions related to motor behavior, which is associated with an improvement in motor symptoms [50]. Levodopa also helps normalize the excessive beta band connectivity observed in PD patients, enhancing beta band modulation dynamics crucial for movement timing and synchronization [52]. Furthermore, levodopa alters functional connectivity within the basal ganglia motor circuit. However, we did not find any alteration in the basal ganglia regions. This result may be due to the sample size or to the early stage of patients’ disease. Our findings of increased beta band connectivity in the frontal lobe of PD patients, in the absence of medication, underscore the importance of beta rhythms in the pathophysiology of PD and highlight the potential therapeutic effects of levodopa in normalizing these connectivity patterns. We also found alteration in occipital lobe and insula, characterized by a lower central role of these regions within the brain network. Studies based on functional connectivity showed that the insula, along with the occipital cortex, exhibits altered connectivity patterns in PD. Oh et al., found that these changes are linked to both motor and cognitive dysfunctions. Indeed, the insula is known for its role in integrating sensory, emotional, and motor information, and the authors suggested that its involvement, together with the occipital regions, may be associated with alterations in dopaminergic modulation [53]. All together, these findings can help define a pattern of altered brain connectivity that affects the entire cerebral network. Regarding the differences in results observed between the two sexes, the findings confirmed in the male PD population are consistent with previous studies [54,55]. It should be noted that the effect of sex as a confounding variable is still under investigation in the literature, and not all studies report clear and consistent results [54]. However, it is common to observe alterations exclusively in the male population in frontal lobe areas, particularly those belonging to motor regions [55,56]. It should be noted, however, that when dividing the population by sex, the number of female participants was significantly smaller compared to males, so a difference in the results could also be driven by this factor.

It should be noted that only some of these alterations have shown a significant correlation with motor impairment. In fact, the frontal lobe is the only analyzed region that has shown both a significant alteration compared to healthy controls and a strong correlation with the UPDRS. These results, also confirmed by previous studies on PD, underline the importance of the frontal lobe in this condition [57,58,59,60,61,62,63]. Furthermore, our study underscores the importance of frequency-specific analyses in PD pathology. In particular, the significant correlation between the frontal lobe in beta band and the UPDRS-III scores, once again, suggest the importance on beta activity alterations in PD. Coherently, PD patients reported higher EC values of frontal lobe in beta band and the correlation indicates that the higher the EC value, the higher the motor impairment as measured through UPDRS-III. The distinct patterns observed in the alpha and beta bands suggest differential impacts on cortical idling and active cognitive/motor processes, offering insights into the disease’s multifaceted manifestations.

Some limitations must be acknowledged to interpret these findings accurately. Given the exploratory nature of this study, it was not possible to identify a specific parameter for conducting a power analysis to determine the optimal sample size. Therefore, the sample size was determined by referencing similar studies in the literature [8]. Furthermore, the data used in the manuscript were not deposited in a public repository at the time of its publication.

In conclusion, the use of eigenvector centrality not only reinforces previous findings of widespread network alterations in PD but also enhances the understanding of how these changes correlate with disease severity and progression. By offering a more comprehensive perspective on brain network topology, EC serves as a valuable metric for investigating complex neurological conditions like Parkinson’s disease, potentially guiding future research and clinical interventions.

## 5. Conclusions

This study underscores the utility of EC in revealing significant topological alterations in brain connectivity in PD. Our findings demonstrate that these alterations are not confined to motor-related regions but also involve areas such as the frontal and occipital lobes, which are crucial for cognitive functions. The strong correlation between EC in the frontal lobe and motor impairment, as measured by the UPDRS, highlights the relevance of EC as a biomarker for disease severity. Moreover, the observed topological changes in the occipital lobe and insula further emphasize the widespread impact of PD on brain networks.

In conclusion, EC provides a robust framework for understanding the complex network alterations in PD. By capturing the influence and connectivity of brain regions, this measure offers a global perspective on brain network dynamics, potentially informing both diagnosis and therapeutic strategies for PD.

## Data Availability

Data will be available on https://www.ebrains-italy.eu/. The data is expected to be accessible on 1 September 2025.

## Supplementary Materials

The following supporting information can be downloaded at: https://www.mdpi.com/article/10.3390/jcm14031020/s1. Table S1: Detailed information on patients with Parkinson’s disease. The table includes age, disease duration in months and Hoehn and Yahr (H&Y) scale score for each participant; Figure S1. Correlation analysis. The figure displays correlations that were significant before false discovery rate correction.

## References

[1] Pagano G., Niccolini F., Politis M. (2016). Imaging in Parkinson’s Disease. Clin. Med..

[2] Li W., Lao-Kaim N.P., Roussakis A.-A., Martín-Bastida A., Valle-Guzman N., Paul G., Soreq E., Daws R.E., Foltynie T., Barker R.A. (2020). Longitudinal Functional Connectivity Changes Related to Dopaminergic Decline in Parkinson’s Disease. Neuroimage Clin..

[3] Sunwoo M.K., Cha J., Ham J.H., Song S.K., Hong J.Y., Lee J., Sohn Y.H., Lee P.H. (2015). Olfactory Performance and Resting State Functional Connectivity in Non-demented Drug Naive Patients with Parkinson’s Disease. Hum. Brain Mapp..

[4] Manza P., Zhang S., Li C.R., Leung H. (2016). Resting-state Functional Connectivity of the Striatum in Early-stage P Arkinson’s Disease: Cognitive Decline and Motor Symptomatology. Hum. Brain Mapp..

[5] Lopes da Silva F. (2013). EEG and MEG: Relevance to Neuroscience. Neuron.

[6] Polverino A., Troisi Lopez E., Minino R., Liparoti M., Romano A., Trojsi F., Lucidi F., Gollo L., Jirsa V., Sorrentino G. (2022). Flexibility of Fast Brain Dynamics and Disease Severity in Amyotrophic Lateral Sclerosis. Neurology.

[7] Bočková M., Rektor I. (2019). Impairment of Brain Functions in Parkinson’s Disease Reflected by Alterations in Neural Connectivity in EEG Studies: A Viewpoint. Clin. Neurophysiol..

[8] Boon L.I., Geraedts V.J., Hillebrand A., Tannemaat M.R., Contarino M.F., Stam C.J., Berendse H.W. (2019). A Systematic Review of MEG-based Studies in Parkinson’s Disease: The Motor System and Beyond. Hum. Brain Mapp..

[9] Dubbelink K.T.E.O., Stoffers D., Deijen J.B., Twisk J.W.R., Stam C.J., Hillebrand A., Berendse H.W. (2013). Resting-State Functional Connectivity as a Marker of Disease Progression in Parkinson’s Disease: A Longitudinal MEG Study. Neuroimage Clin..

[10] Bullmore E., Sporns O. (2009). Complex Brain Networks: Graph Theoretical Analysis of Structural and Functional Systems. Nat. Rev. Neurosci..

[11] Van Wijk B.C.M., Stam C.J., Daffertshofer A. (2010). Comparing Brain Networks of Different Size and Connectivity Density Using Graph Theory. PLoS ONE.

[12] Binnewijzend M.A.A., Adriaanse S.M., Van der Flier W.M., Teunissen C.E., De Munck J.C., Stam C.J., Scheltens P., Van Berckel B.N.M., Barkhof F., Wink A.M. (2014). Brain Network Alterations in Alzheimer’s Disease Measured by Eigenvector Centrality in FMRI Are Related to Cognition and CSF Biomarkers. Hum. Brain Mapp..

[13] Cao F., Guan X., Ma Y., Shao Y., Zhong J. (2020). Altered Functional Network Associated with Cognitive Performance in Early Parkinson Disease Measured by Eigenvector Centrality Mapping. Front. Aging Neurosci..

[14] Fraschini M., Hillebrand A., Demuru M., Didaci L., Marcialis G.L. (2014). An EEG-Based Biometric System Using Eigenvector Centrality in Resting State Brain Networks. IEEE Signal Process. Lett..

[15] Lohmann G., Margulies D.S., Horstmann A., Pleger B., Lepsien J., Goldhahn D., Schloegl H., Stumvoll M., Villringer A., Turner R. (2010). Eigenvector Centrality Mapping for Analyzing Connectivity Patterns in FMRI Data of the Human Brain. PLoS ONE.

[16] Lorenzini L., Ingala S., Collij L.E., Wottschel V., Haller S., Blennow K., Frisoni G., Chételat G., Payoux P., Lage-Martinez P. (2023). Eigenvector Centrality Dynamics Are Related to Alzheimer’s Disease Pathological Changes in Non-Demented Individuals. Brain Commun..

[17] Sorriso A., Sorrentino P., Rucco R., Mandolesi L., Ferraioli G., Franceschini S., Ambrosanio M., Baselice F. (2019). An Automated Magnetoencephalographic Data Cleaning Algorithm. Comput. Methods Biomech. Biomed. Eng..

[18] Troisi Lopez E., Minino R., Liparoti M., Polverino A., Romano A., De Micco R., Lucidi F., Tessitore A., Amico E., Sorrentino G. (2023). Fading of Brain Network Fingerprint in Parkinson’s Disease Predicts Motor Clinical Impairment. Hum. Brain Mapp..

[19] Baselice F., Sorriso A., Rucco R., Sorrentino P. (2019). Phase Linearity Measurement: A Novel Index for Brain Functional Connectivity. IEEE Trans. Med. Imaging.

[20] Sorrentino P., Ambrosanio M., Rucco R., Baselice F. (2019). An Extension of Phase Linearity Measurement for Revealing Cross Frequency Coupling among Brain Areas. J. Neuroeng. Rehabil..

[21] Sorrentino P., Ambrosanio M., Rucco R., Cabral J., Gollo L.L., Breakspear M., Baselice F. (2022). Detection of Cross-Frequency Coupling Between Brain Areas: An Extension of Phase Linearity Measurement. Front. Neurosci..

[22] Gelb D.J., Oliver E., Gilman S. (1999). Diagnostic Criteria for Parkinson Disease. Arch. Neurol..

[23] Hoehn M.M., Yahr M.D. (1967). Parkinsonism: Onset, Progression, and Mortality. Neurology.

[24] Emre M., Aarsland D., Brown R., Burn D.J., Duyckaerts C., Mizuno Y., Broe G.A., Cummings J., Dickson D.W., Gauthier S. (2007). Clinical Diagnostic Criteria for Dementia Associated with Parkinson’s Disease. Mov. Disord..

[25] McKeith I.G., Boeve B.F., Dickson D.W., Halliday G., Taylor J.-P., Weintraub D., Aarsland D., Galvin J., Attems J., Ballard C.G. (2017). Diagnosis and Management of Dementia with Lewy Bodies: Fourth Consensus Report of the DLB Consortium. Neurology.

[26] Goetz C.G., Tilley B.C., Shaftman S.R., Stebbins G.T., Fahn S., Martinez-Martin P., Poewe W., Sampaio C., Stern M.B., Dodel R. (2008). Movement Disorder Society-sponsored Revision of the Unified Parkinson’s Disease Rating Scale (MDS-UPDRS): Scale Presentation and Clinimetric Testing Results. Mov. Disord..

[27] Rombetto S., Granata C., Vettoliere A., Russo M. (2014). Multichannel System Based on a High Sensitivity Superconductive Sensor for Magnetoencephalography. Sensors.

[28] Granata C., Vettoliere A., Russo M. (2006). Improved Superconducting Quantum Interference Device Magnetometer for Low Cross Talk Operation. Appl. Phys. Lett..

[29] Liparoti M., Troisi Lopez E., Sarno L., Rucco R., Minino R., Pesoli M., Perruolo G., Formisano P., Lucidi F., Sorrentino G. (2021). Functional Brain Network Topology across the Menstrual Cycle Is Estradiol Dependent and Correlates with Individual Well-being. J. Neurosci. Res..

[30] Fraschini M., Demuru M., Crobe A., Marrosu F., Stam C.J., Hillebrand A. (2016). The Effect of Epoch Length on Estimated EEG Functional Connectivity and Brain Network Organisation. J. Neural Eng..

[31] Gross J., Baillet S., Barnes G.R., Henson R.N., Hillebrand A., Jensen O., Jerbi K., Litvak V., Maess B., Oostenveld R. (2013). Good Practice for Conducting and Reporting MEG Research. Neuroimage.

[32] Sorrentino P., Rucco R., Baselice F., De Micco R., Tessitore A., Hillebrand A., Mandolesi L., Breakspear M., Gollo L.L., Sorrentino G. (2021). Flexible Brain Dynamics Underpins Complex Behaviours as Observed in Parkinson’s Disease. Sci. Rep..

[33] Oostenveld R., Fries P., Maris E., Schoffelen J.-M. (2011). FieldTrip: Open Source Software for Advanced Analysis of MEG, EEG, and Invasive Electrophysiological Data. Comput. Intell. Neurosci..

[34] De Cheveigné A., Simon J.Z. (2007). Denoising Based on Time-Shift PCA. J. Neurosci. Methods.

[35] Barbati G., Porcaro C., Zappasodi F., Rossini P.M., Tecchio F. (2004). Optimization of an Independent Component Analysis Approach for Artifact Identification and Removal in Magnetoencephalographic Signals. Clin. Neurophysiol..

[36] Gong G., He Y., Concha L., Lebel C., Gross D.W., Evans A.C., Beaulieu C. (2009). Mapping Anatomical Connectivity Patterns of Human Cerebral Cortex Using in Vivo Diffusion Tensor Imaging Tractography. Cereb. Cortex.

[37] Tzourio-Mazoyer N., Landeau B., Papathanassiou D., Crivello F., Etard O., Delcroix N., Mazoyer B., Joliot M. (2002). Automated Anatomical Labeling of Activations in SPM Using a Macroscopic Anatomical Parcellation of the MNI MRI Single-Subject Brain. Neuroimage.

[38] Zaki M., Meira W. (2014). Data Mining and Analysis: Fundamental Concepts and Algorithms.

[39] Negre C.F.A., Morzan U.N., Hendrickson H.P., Pal R., Lisi G.P., Loria J.P., Rivalta I., Ho J., Batista V.S. (2018). Eigenvector Centrality for Characterization of Protein Allosteric Pathways. Proc. Natl. Acad. Sci. USA.

[40] Newman M.E.J. (2008). The Mathematics of Networks. New Palgrave Encycl. Econ..

[41] Benjamini Y., Hochberg Y. (1995). Controlling the False Discovery Rate: A Practical and Powerful Approach to Multiple Testing. J. R. Stat. Soc. Ser. B (Methodol.).

[42] Sreenivasan K., Mishra V., Bird C., Zhuang X., Yang Z., Cordes D., Walsh R.R. (2019). Altered Functional Network Topology Correlates with Clinical Measures in Very Early-Stage, Drug-Naive Parkinson’s Disease. Park. Relat. Disord..

[43] Li C., Huang B., Zhang R., Ma Q., Yang W., Wang L., Wang L., Xu Q., Feng J., Liu L. (2017). Impaired Topological Architecture of Brain Structural Networks in Idiopathic Parkinson’s Disease: A DTI Study. Brain Imaging Behav..

[44] Huang L.-C., Wu P.-A., Lin S.-Z., Pang C.-Y., Chen S.-Y. (2019). Graph Theory and Network Topological Metrics May Be the Potential Biomarker in Parkinson’s Disease. J. Clin. Neurosci..

[45] Pourmotahari F., Tabatabaei S.M., Borumandnia N., Khadembashi N., Olazadeh K., Alavimajd H. (2024). A Study Over Brain Connectivity Network of Parkinson’s Patients, Using Nonparametric Bayesian Model. Basic Clin. Neurosci..

[46] Mijalkov M., Volpe G., Pereira J.B. (2022). Directed Brain Connectivity Identifies Widespread Functional Network Abnormalities in Parkinson’s Disease. Cereb. Cortex.

[47] Zhou Z., Yan Y., Gu H., Sun R., Liao Z., Xue K., Tang C. (2024). Dopamine in the Prefrontal Cortex Plays Multiple Roles in the Executive Function of Patients with Parkinson’s Disease. Neural Regen. Res..

[48] Tang C., Sun R., Xue K., Wang M., Liang S., Kambey P.A., Shi M., Wu C., Chen G., Gao D. (2024). Distinct Serum GDNF Coupling with Brain Structural and Functional Changes Underlies Cognitive Status in Parkinson’s Disease. CNS Neurosci. Ther..

[49] Göttlich M., Münte T.F., Heldmann M., Kasten M., Hagenah J., Krämer U.M. (2013). Altered Resting State Brain Networks in Parkinson’s Disease. PLoS ONE.

[50] Chung J.W., Burciu R.G., Ofori E., Coombes S.A., Christou E.A., Okun M.S., Hess C.W., Vaillancourt D.E. (2018). Beta-Band Oscillations in the Supplementary Motor Cortex Are Modulated by Levodopa and Associated with Functional Activity in the Basal Ganglia. Neuroimage Clin..

[51] Levy R., Hutchison W.D., Lozano A.M., Dostrovsky J.O. (2002). Synchronized Neuronal Discharge in the Basal Ganglia of Parkinsonian Patients Is Limited to Oscillatory Activity. J. Neurosci..

[52] Evangelisti S., Pittau F., Testa C., Rizzo G., Gramegna L.L., Ferri L., Coito A., Cortelli P., Calandra-Buonaura G., Bisquoli F. (2019). L-Dopa Modulation of Brain Connectivity in Parkinson’s Disease Patients: A Pilot EEG-FMRI Study. Front. Neurosci..

[53] Oh S.W., Shin N.-Y., Yoon U., Sin I., Lee S.-K. (2020). Shared Functional Neural Substrates in Parkinson’s Disease and Drug-Induced Parkinsonism: Association with Dopaminergic Depletion. Sci. Rep..

[54] Diez-Cirarda M., Gabilondo I., Ibarretxe-Bilbao N., Gómez-Esteban J.C., Kim J., Lucas-Jiménez O., Del Pino R., Peña J., Ojeda N., Mihaescu A. (2021). Contributions of Sex, Depression, and Cognition on Brain Connectivity Dynamics in Parkinson’s Disease. NPJ Park. Dis..

[55] Oltra J., Uribe C., Campabadal A., Inguanzo A., Monté-Rubio G.C., Martí M.J., Compta Y., Valldeoriola F., Junque C., Segura B. (2022). Sex Differences in Brain and Cognition in de Novo Parkinson’s Disease. Front. Aging Neurosci..

[56] De Micco R., Esposito F., di Nardo F., Caiazzo G., Siciliano M., Russo A., Cirillo M., Tedeschi G., Tessitore A. (2019). Sex-related Pattern of Intrinsic Brain Connectivity in Drug-naïve Parkinson’s Disease Patients. Mov. Disord..

[57] Cools R., Miyakawa A., Sheridan M., D’Esposito M. (2010). Enhanced Frontal Function in Parkinson’s Disease. Brain.

[58] Hou Y., Luo C., Yang J., Ou R., Song W., Wei Q., Cao B., Zhao B., Wu Y., Shang H.-F. (2016). Prediction of Individual Clinical Scores in Patients with Parkinson’s Disease Using Resting-State Functional Magnetic Resonance Imaging. J. Neurol. Sci..

[59] Huang T., Tang L., Zhao J., Shang S.A., Chen Y., Tian Y., Zhang Y. (2023). Drooling Disrupts the Brain Functional Connectivity Network in Parkinson’s Disease. CNS Neurosci. Ther..

[60] Maidan I., Nieuwhof F., Bernad-Elazari H., Reelick M.F., Bloem B.R., Giladi N., Deutsch J.E., Hausdorff J.M., Claassen J.A.H., Mirelman A. (2016). The Role of the Frontal Lobe in Complex Walking among Patients with Parkinson’s Disease and Healthy Older Adults: An FNIRS Study. Neurorehabil. Neural Repair.

[61] Schneider L., Seeger V., Timmermann L., Florin E. (2020). Electrophysiological Resting State Networks of Predominantly Akinetic-Rigid Parkinson Patients: Effects of Dopamine Therapy. Neuroimage Clin..

[62] Yadav R., Pal P.K., Ingalhalikar M. (2017). Altered Brain Wiring in Parkinson’s Disease: A Structural Connectome-Based Analysis. Brain Connect..

[63] Yang M., Huang X., Huang L., Cai G. (2023). Diagnosis of Parkinson’s Disease Based on 3D ResNet: The Frontal Lobe Is Crucial. Biomed. Signal Process. Control.

