# Supplementary material for: Analyzing Information Exchange in Parkinson’s Disease via Eigenvector Centrality: A Source-Level Magnetoencephalography Study
Source: J Clin Med. 2025 Feb 5;14(3):1020. doi: 10.3390/jcm14031020 (PMC11818797; doi:10.3390/jcm14031020)
Supplement: Supplementary file 1 [file jcm-14-01020-s001.zip › jcm-3416948-supplementary.pdf]

ID	Age	Disease duration (months)	H&Y
S_01	50	77	2.5
S_02	63	24	2
S_03	60	28	1
S_04	72	72	1
S_05	70	24	2
S_06	68	48	2
S_07	59	24	2
S_08	65	42	2
S_09	74	36	2
S_10	73	96	2.5
S_11	70	44	1
S_12	59	36	2
S_13	51	45	2
S_14	55	72	2.5
S_15	74	36	2.5
S_16	58	6	1
S_17	84	32	2.5
S_18	57	24	1
S_19	51	36	2
S_20	49	48	2
S_21	64	48	2
S_22	78	60	1.5
S_23	63	21	2
S_24	55	51	2.5
S_25	72	96	2.5
S_26	71	36	2
S_27	44	36	1
S_28	69	21	2
S_29	70	26	1.5
S_30	60	10	1
S_31	73	60	2.5
S_32	65	9	1
S_33	70	24	2.5
S_34	64	48	2
S_35	77	42	1
S_36	53	72	2
S_37	61	30	1
S_38	61	48	2.5
S_39	69	12	2
S_40	57	14	1.5
S_41	82	54	2
S_42	83	41	2
S_43	71	12	1

S_44	71	96	2
S_45	72	120	2.5
S_46	61	5	1
S_47	47	24	2

Table S1. Detailed information on patients with Parkinson's disease. The table includes age, disease duration in months and Hoehn and Yahr (H&Y) scale score for each participant.

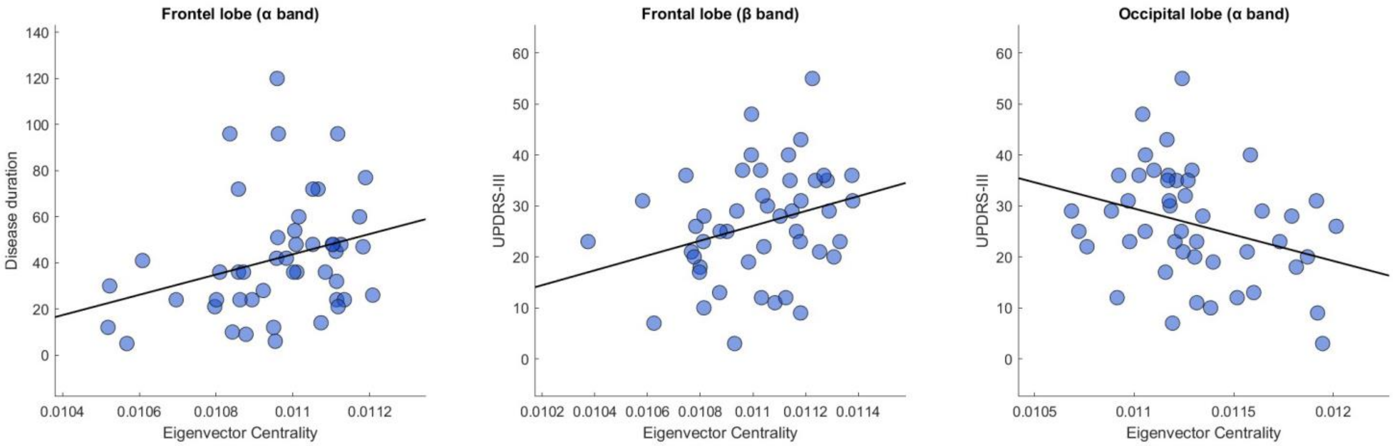

Figure S1. Correlation analysis. The figure displays correlations that were significant before false discovery rate correction.
